# Rule-Based Information Extraction from Free-Text Pathology Reports Reveals Trends in South African Female Breast Cancer Molecular Subtypes and Ki67 Expression

**DOI:** 10.1155/2022/6157861

**Published:** 2022-01-20

**Authors:** Okechinyere J. Achilonu, Elvira Singh, Gideon Nimako, René M. J. C. Eijkemans, Eustasius Musenge

**Affiliations:** ^1^Division of Epidemiology and Biostatistics, School of Public Health, Faculty of Health Sciences, University of the Witwatersrand, Parktown, Johannesburg, South Africa; ^2^National Cancer Registry, National Health Laboratory Service, 1 Modderfontein Road, Sandringham, Johannesburg, South Africa; ^3^Industrialization, Science, Technology and Innovation Hub, African Union Development Agency (AUDA-NEPAD), Johannesburg, South Africa; ^4^Julius Center for Health Sciences and Primary Care, University Medical Center, Utrecht University, Utrecht, Netherlands

## Abstract

Clinical information on molecular subtypes and the Ki67 index is critical for breast cancer (BC) prognosis and personalised treatment plan. Extracting such information into structured data is essential for research, auditing, and cancer incidence reporting and underpins the potential for automated decision support. Herewith, we developed a rule-based natural language processing algorithm that retrieved and extracted important BC parameters from free-text pathology reports towards exploring molecular subtypes and Ki67-proliferation trends. We considered malignant BC pathology reports with different free-text narrative attributes from the South African National Health Laboratory Service. The reports were preprocessed and parsed through the algorithm. Parameters extracted by the algorithm were validated against manually extracted parameters. For all parameters extracted, we obtained accurate annotations of 83-100%, 93-100%, 91-100%, and 92-100% precision, recall, *F*_1_-score, and kappa, respectively. There was a significant trend in the proportion of each molecular subtype by patient age, histologic type, grade, Ki67, and race. The findings also showed significant association in the Ki67 trend with hormone receptors, human epidermal growth factors, age, grade, and race. Our approach bridges the gap between data availability and actionable knowledge and provides a framework that could be adapted and reused in other cancers and beyond cancer studies. Information extracted from these reports showed interesting trends that may be exploited for BC screening and treatment resources in South Africa. Finally, this study strongly encourages the implementation of a synoptic style pathology report in South Africa.

## 1. Introduction

Breast cancer (BC) is a complex and heterogeneous disease and remains the most commonly diagnosed malignancy among women in South Africa [1]. The prognosis of this disease depends on several biological and clinical features, including oestrogen and progesterone (ER and PR) receptors, human epidermal growth factor (HER2) receptor, Ki67 proliferation index, histologic type, and tumour grade [2–5]. This histopathology information forms the basis for a patient's optimal treatment decisions, described in a pathology report. Thus, a cancer pathology report provides substantive and valuable information on these features mentioned earlier, representing the clinical condition of a cancer patient [6]. Nonetheless, South African pathology reports are not structured or synoptic. The synoptic report uses a checklist-style to report all the compulsory parameters, following a set standard or format [1, 7, 8]. An accurate histopathology report is critical in providing essential prognostic and predictive parameters required for more streamlined patient care. Besides the direct use of this information in the health care system, pathology reports are essential for research, audit, and cancer incidence reporting [9].

The South African National Health Laboratory Services (NHLS) employs free-text narrative-style cancer pathology reporting. This type of report lacks a structural framework and may be presented with several errors, including semantic ambiguity, spelling errors, improper grammar and literary style, and local language [10]. In addition, challenges such as comprehensiveness, leading to heterogeneity within a reporting institution, are frequently encountered with narrative-style free-text reports [7]. The South African NHLS employs data coders to manually extract valuable information and translate the information based on clinical rules [11]. Manual extraction of information from free-text reports is expensive and time-consuming [12]. An automated retrieval system from pathology reports can enable expedient and timeous preparation of a multicentre and population-level study, which will result in significant cost savings and the creation of consistent pathology reporting at the national level.

Text mining (TM) has emerged as a computational technique to timely and accurately transform pathology reports into a structured data representation. TM leverages methods from natural language processing (NLP), knowledge discovery, and machine learning (ML) and has been successfully applied towards named entity recognition, information retrieval, information extraction (IE), and document classification [13]. IE is a relevant branch of NLP concerned with extracting structured data from unstructured data based on predefined information. ML and rule-based approaches are commonly used for clinical IE from unstructured data. Over the years, supervised ML techniques have been widely applied for clinical IE and have shown efficiency and effectiveness with different medical data [14]. The rule-based approach consists of a set of rules for matching patterns and performing actions in a text [14]. Several NLP algorithms, including MedLee, cTAKES, and MetaMap for clinical IE extraction, have been developed [14, 15]. However, challenges are encountered in the implementation of these tools due to institution-specific reporting styles, which leads to a lack of generalization in other settings [16].

In cancer research, IE with rule-based methods has been used to extract critical prognostic features from prostate and skin cancer pathology reports [17], as well as specimens and their related findings from free-text surgical pathology report [18]. Clinically useful information from patients with hepatocellular carcinoma [19], among others, has also been reported [20–22]. A few studies have used a regular expression (*Regex*) rule-based approach to extract essential parameters from BC pathology reports. Reference [23] used the *Regex* function to retrieve and analyse PR, ER, and HER2 characteristics in primary and recurrent BC. These authors also extended their study to extract the same parameter from metastatic breast tumours [24]. Their proposed programmes achieved high sensitivity in both studies. The difference between our approach and the above studies is that we analysed all the carcinoma cases in the database and did not programme the syntax specific to carcinoma type or stage. In other words, all carcinoma cases were considered because of the aim of this study. Another notable difference is that we went beyond just showing summary statistics of these parameters to (i) extracting other clinically relevant BC parameters and (ii) exploring the association between the molecular subtypes, Ki67 overexpression, and other BC parameters.

To our knowledge, this is the first study to use a rule-based NLP approach to extract information from pathology reports in South Africa and elsewhere in Africa. Earlier research by [2] studied racial comparison of receptor-defined BC in South African and Namibian Women between 2009 and 2011. The information used in their study was extracted from the South Africa National Cancer Registry and the Namibia Cancer Hospital. However, the algorithm employed in their extraction processes and its function was neither mentioned nor described nor made available for future studies and reproducibility of their study.

In our recent study [25], we developed an automated model for free-text pathology report identification and classification. The study approach created structured data with several parameters. For each parameter, the technique assigns “1” to a case if the parameter is found in the pathology report; otherwise, “0.” We identified BC parameters that significantly contribute to the discrimination of benign and malignancy classes. Following this previous study, we aimed to extract these key clinically relevant parameters and their corresponding values to assess the trend of BC molecular subtypes and the Ki67 proliferation index. 1This study was aimed at creating structured data comprising important BC prognostic parameters for research purposes. The secondary aim was to explore the trend of BC molecular subtypes and the Ki67 proliferation index in women diagnosed with BC between 2011 and 2019. Our study was aimed at answering the following questions using the concept of *Regex* matching rule-based approach:

- How should a target parameter and its corresponding values be standardised given several ways of representation in a free-text pathology report?
- Does the pathology report contain all the target parameters and corresponding values? If yes, to what degree can our automated approach match all the patterns and accurately extract these parameters and their associated values?
- Has there been consistency in the comprehensiveness and completeness of BC pathology reporting over the year?
- What is the trend of the target parameters and their association with other known parameters?

We defined parameters and their associated values to guide the extraction to answer these questions. The BC pathology reports were parsed using the *Regex* matching functions, automatically transforming the reports into structured data that can be examined and queried based on the target parameters. Our approach bridges the gap between data availability and actionable knowledge and provides a framework that could be adapted and reused in other cancers and beyond cancer studies.

The trend analysis was done on the molecular subtype and Ki67 in relation to other key features such as age, race, grade, laterality, and histological type of the tumour. This may also be considered validation and affirmation of the authenticity and usefulness of our developed algorithm. This is because if the *Regex* matching algorithm is efficient, then the trend in these features will be comparable to previous studies. Fortunately, several studies have been published on the trend in BC molecular subtypes, making the comparison easier. To reduce biases and improve the generalizability of our findings, we attempted to follow published criteria for these study parameters. Overall, the sample size used in our study was sufficient to produce a reliable trend in these BC prognostic parameters, and inferences can be made from this study without equivocation.

## 2. Materials and Methods

### 2.1. Study Data

**Figure 1:**
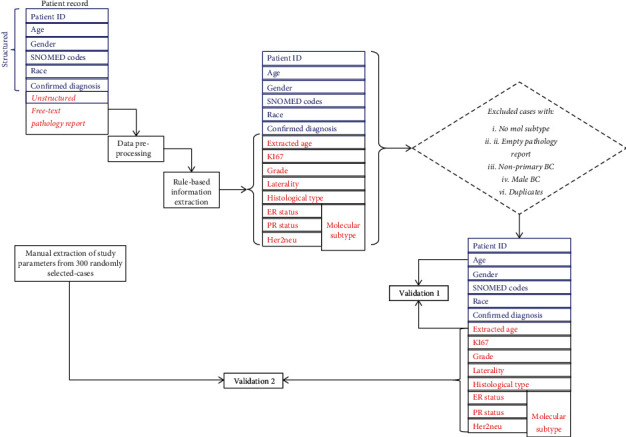
Overview of case study identification architecture. Protocol for extracting each study parameter was developed and used as a guide to reduce the chances of extracting noise in the study data. Each patient pathology report was parsed through the extraction process to extract the target parameters, which were combined with the structured data to create a complete patient profile. The profile was assessed for eligibility criteria to check whether or not a case is qualified for inclusion in this study.

**Figure 2:**
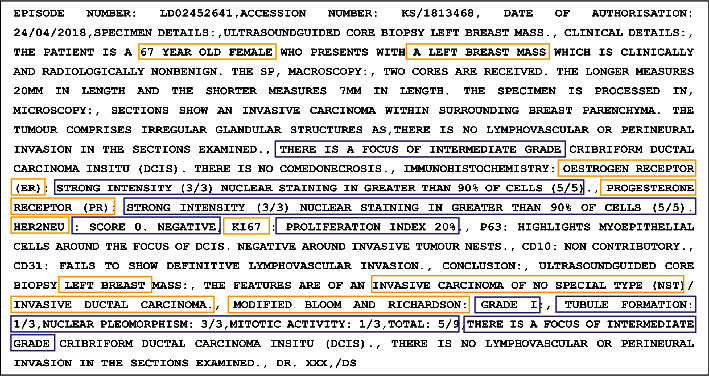
A sample of pathology report illustrating the target parameters (gold box) and their corresponding values (purple box). Patient age: 67, laterality: left breast, tumour grade: 5/9 = I, ER: 8/8 = positive, PR: 8/8 = positive, HER2: 0 = negative, Ki67: ≥14, and histological type: IDC.

This retrospective and descriptive study involved BC cases and was approved by the Human Research Ethics Committee (Medical) of the University of the Witwatersrand, Johannesburg, South Africa (M1911131). We obtained BC pathology reports in pure text form (between 2008 and 2019) from the Corporate Data Warehouse of NHLS (NHLS-CDW). The NHLS is the largest diagnostic pathology service laboratory in South Africa, with a network of approximately 226 pathology laboratories. It provides clinical support services to over 80% of the population through its countrywide diagnostic laboratories [26]. Each patient's data consists of both structured and unstructured information, including the SNOMED code (for morphology and topography), confirmed diagnosis, age, race, and the pathology report (Figure 1). The SNOMED code is a string value used in most tumour registries to represent health terminologies [27]. The values mapped with the international classification of disease (ICD-03) for semantic interoperability. ICD-03 is the lingua franca of pathologists, which is globally used within tumour registries [28].  Figure 2 shows a sample of the free-text narrative-style pathology report used in this study. This report describes a malignant breast tumour containing the target study parameters (including ER, PR, Ki67, and HER2) and their corresponding values and other features that are not of interest to this study. As defined previously, a synoptic report would not contain all the information in Figure 2. An example of a synoptic style report is shown in this study by [8]. The synoptic report illustrated in the study in [8] is specific, and the parameters are mentioned followed by their corresponding values, which improves consistency over the free-style report.

### 2.2. Retrieval of Malignant Breast Carcinoma Cases and Data Preprocessing

We initially started with all the pathology reports to ensure that we did not miss any cases that met the eligibility criteria for this study. Information retrieval was conducted to categorise each report in the database as relevant or irrelevant to the study objectives [15]. The SNOMED codes were mapped to the ICD-03 to extract malignant cases. This was done using the *Regex* function in the *R* software. *Regex* is a rule-based NLP tool defined as an algebraic notation for pattern searching in a corpus of texts [29]. For a SNOMED code denoted by “M-85203,” the first four values represent the morphology. In contrast, the last value represents the behaviour. A malignancy class was created, and a “*stringreplaceallfunction*” was used to search for patterns in the SNOMED codes ending with behaviour values 2, 3, and 6 to populate this class. Only cases with these SNOMED code values were retrieved for this study. The pathology reports were preprocessed by removing excess spaces and characters, including asterisks, colon, and parenthesis.

**Figure 3:**
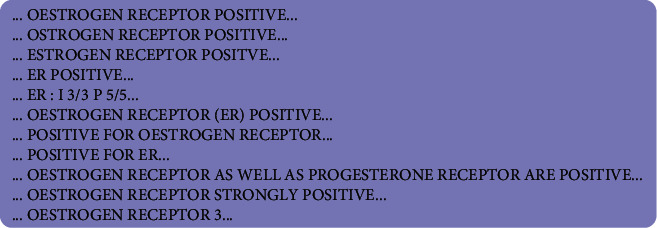
A few examples of variations identified in the NHLS pathology reports in referring to ER parameter and linking it to its corresponding score or values.

Identifying the parameters of interest and their reporting style in a text is the basis for the application of TM [30]. The named entity recognition consists of recognising and normalising the parameters of interest. We reviewed studies on BC clinical parameters. We identified names, synonyms, and categories used to denote the parameters of interest [4, 31–35]. These studies mentioned above were used to construct a dictionary of features to be extracted and to guide our extraction process. We searched and identified different variants in the reporting style of these parameters in the pathology reports. To standardise the parameters and their corresponding values, we categorised all the reporting variants into structured name entities for each study parameter and did the same for their values. Although the format of the reports is poorly standardised and inconsistent, our preprocessing approach was able to reduce the variation to optimise the searching process and enabled a broader extraction of the study parameters.  Figure 3 is an example of 11 reporting style variants for positive ER score identified in the pathology reports.

### 2.3. Extraction of Important Study Parameters

We programmed the *Regex* function to search within the free-text report and extract phrases specific to each study parameter. In other words, the process reduced the text length of the reports while retaining phrases that contain the target parameters. This stage of extraction could be likened to text summarisation [36]. For each phrase retrieved, we examined the presence of the target parameter and its corresponding values or scores. Reports containing evidence of each of these parameters were retained for further analysis. The extraction process was not accomplished with just a single run of the *Regex* function. For instance, in the extraction of the “Ki67” parameter with its value for a patient, we programmed the first run to summarise the report while targeting the term “Ki67.” In the second run, we further reduced the search to 17 characters to remove irrelevant words while retaining the target and values. The *Regex* function was set to look into the previous-run extraction, which contains the term “Ki67,” match, and retrieve any 0-4 digit with or without percentage symbol (%). The fourth run addressed a scenario where the parameter was already categorised in the report by the pathologist. Hence, we implemented the *Regex* function to also look into the second run, targeting the term “Ki67,” and extract where the pathologist was specific to mention “positive,” “negative,” “low,” or “high.” Several iterations were run to synthesise the extracted categories and values of this parameter. In the end, the final extracted columns were combined to form structured data. The pseudo- and programming codes illustrating “Ki67” extraction are shown in Pseudocodes 1 and 2.

Various challenging scenarios were experienced during the retrieval and extraction processes. First of all, the lack of a standard structured format of reporting the parameters proved to be the major challenge encountered in the extraction. Several name variations were often used to denote a parameter, some of which are short forms, and some are longer forms. For example, the human epidermal growth factor was written as “HER2NUE,” “HER2-NUE,” “HER2/NUE,” “HER2(NUE),” “HERNUE,” “HER2,” “HER,” “CERB,” “CERB-B2,” “CERBB2,” “CERBB,” and “C-erb2/HER2.” In addition, we observed more complex variations in linking some parameters to their associated scores or values. Therefore, for our system to complete the extraction of some parameters, more than 10 to 50 extraction steps may be carried out, depending on how the parameter was reported. In addition, we identified several spelling errors of the parameters relevant to the study, which may affect their extraction process. To address this, we identified and reexamined cases where the extraction process failed and recoded these parameters or their values to improve on the number of extractable cases or reports from the data set. Nonetheless, the algorithm extracted more than 98% of all extractable parameters before this recoding step.

The description of each extracted parameter is shown in the supplementary section. The statuses of ER, PR, and HER2 (for each case) were identified and combined to create the molecular subtype parameter (Table 1), as described in a study by [37]. In the end, we defined completeness of reporting for each case based on the presence of the molecular subtypes. Exclusion criteria were defined after the completion of the parameter extraction based on the scope of this study (Figure 1). Cases without molecular subtype information were excluded from further study. The patient episode numbers were used to exclude duplicate cases in the study data. These duplicates were compared with the main data to observe any variation between the two data sets. We observed that almost all the patients studied have two exact copies of the same information in the NHLS-CDW database. However, about 18 patients had more than two pathology reports, which contained disparate information. These 18 patients' information was compared with their records in the main data set and used to replace the missing information in the main data set. We subsequently excluded cases with empty pathology reports from the study and male BC cases. At this stage, the final data set consisting of 9669 cases with the complete report was retained for further analysis. The extraction procedure in this study was done using both simple and extended *Regex*′s language implemented in *R* software. The full details of the *Regex* syntax have been deposited in the GitHub platform for the adaptation and reproducibility of this study (https://github.com/KechJay/Information-retrieval-and-extraction-BC).

ER: oestrogen receptor; PR: progesterone receptor; HER2: human epidermal factor; TNBC: triple negative breast cancer; HER2-OE: Her2 overexpression.

### 2.4. Validation of Extracted Information

As shown in Figure 1, only the patient age and the histological type were manually annotated by the NHLS-CDW data coders as structured in the retrieved data. They were used to validate the result of our extraction for these two parameters. To further validate the information extracted for this study, we performed a manual extraction of 300 pathology records randomly sampled from the final study data set. The manual review was considered a gold standard. Two annotators were trained on the parameters and the range of linking values they should extract to create this data set. Developed guidelines for the annotation task was given to them. An expert rater resolved the differences between the annotations by the two raters. The high agreement level between the manual raters could be attributed to the developed guideline and the small sample size. Interannotator agreement (IAA) studies were conducted to assess the agreement between the manually extracted data and machine-extracted data [38]. In the context of this study, IAA relates to the extent to which manual and machine-assisted extractions assign the same patient score for each parameter. For the categorical parameters, IAA was estimated with Cohen's kappa coefficient (*k*), a pairwise reliability measure for nominal data [39]. *k* is defined by

(1) $$\begin{array}{c} \text{kappa }\left(k\right)=\frac{P_{0}-P_{e}}{1-P_{e}}, \end{array}$$

where *P*_0_ (accuracy) is the relative observed agreement between the manual and machine-assisted extraction and *P*_*e*_ is the expected probability chance agreement. Also, evaluation of our approach was made with precision, recall, and *F*_1_-score measurements. Precision measures the number of correct parameter values retrieved by the machine divided by all retrieved parameter values. Recall measures the number of correct parameter values retrieved by the machine divided by all correct parameter values, and the *F*_1_-score is the weighted harmonic mean of precision and recall. These measures are defined as follows:

(2) $$\begin{array}{c} \text{Precision }\left(P\right)=\frac{\text{TP}}{\text{TP}+\text{FP}},\\ \text{Recall }\left(R\right)=\frac{\text{TP}}{\text{TP}+\text{FN}},\\ F_{1}‐\text{score}=2\frac{PR}{P+R}, \end{array}$$

where TP is true positive, TN is true negative, FP is false positive, and FN is false negative.

For the age parameter, we used the intraclass correlation coefficient (ICC), a method for continuous parameter assessment [38]. In this context, ICC relates to the proportion of variance assignable to the annotation of patient age by manual and machine-assisted extraction. We used the two-way mixed model ICC type to estimate the average score as defined by

(3) $$\begin{array}{c} \text{IC}{\text{C}}_{A,K}=\frac{MS_{B}-MS_{E}}{MS_{B}+\left(MS_{R}-MS_{E}\right)/n}, \end{array}$$

where *n* is the number of observations, and the mean squares are based on the analysis of variance table as described in [38, 40]. The interannotator agreement analysis was done using the“*irrpackage*^”^ and in *R* software.

### 2.5. Statistical Analysis

Descriptive analysis was conducted, and the result was displayed in data visualisation and summary statistics. Some parameters such as “race” and “histological grade” included in this study contain missing information due to the heterogeneity or incompleteness associated with the free-style reporting. The missingness varies per parameter, depending on the way it was reported. For instance, the patient race has the highest level of missingness (≈43%); this is because patient race is underreported in the pathology reports. However, this information is usually captured in the patient's hospital records. Unlike “race,” the patient's age is well reported in the pathology report, leading to a few missingness for this variable.

We replaced the missing values using the missForest ML imputation technique that has been shown to be efficient and effective in imputing different types of data [41, 42]. Analysis was carried out with both the imputed and the complete case (CC) data. However, the CC analysis was reported in the supplementary section. Multinomial Logistic regression (MLR) was performed [43] to evaluate the association between the molecular subtypes and the parameters. MLR is an extension of binary logistic regression to predict a nominal response variable. The molecular subtypes, which is the response variable, has four categories. The probability that a patient diagnosed with Luminal A was used as the reference category, and the other *k* − 1 categories were separately used to regress against the reference category. This model has been applied in similar studies and has shown effectiveness in describing the parameter of interest [44, 45]. We modelled the probability of each outcome as

(4) $$\begin{array}{c} P\left(Y=j\right)=\frac{e^{\beta_{j}X_{i}}}{1+\sum_{j=1}^{j}e^{\beta_{j}X_{i}}}, \end{array}$$

where *β*_*j*_ is the set of regression coefficients associated with outcome *j* and *X*_*i*_ is each extracted parameter associated with observation *i*. We also defined a binary model, where *Y* = 2 classes in equation (4), for the Ki67 pattern analysis. Complete information for the Ki67 was extracted from the study data and was used to assess its relationship with other study parameters.

## 3. Results

Our *Regex* matching algorithm identified a total of 9669 cases that met all the eligibility criteria for this study. The constructed data contains eight parameters with their corresponding values. The evaluation performance of the extracted data based on our algorithm and the manually extracted data is shown in Table 2. Overall, we obtained accurate annotations ranging from 83-100%, 93-100%, 91-100%, and 92-100% for precision, recall, *F*_1_-score, and kappa, respectively. The algorithm achieved the highest percentage annotation for histological type and HER2 (99-100%), followed by laterality. On the other hand, we obtained a lower performance for tumour grade I and Ki67 < 14, which are the categories with lower frequencies. The evaluation of the hormone receptor extractions (often reported with long and complex sentences), specifically PR, yielded up to 99% precision and recall. For the categorical variables, we observed that errors were associated with complexity in linking the target parameter to its corresponding values, more pronounced in categories with lower frequencies.

**Figure 4:**
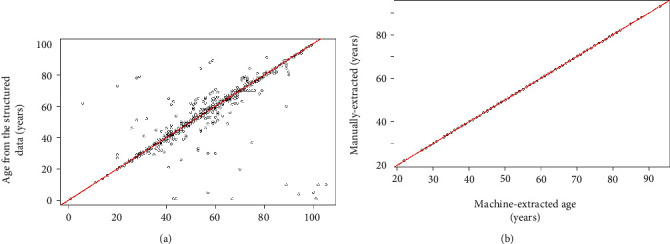
Correlation between extracted age based on *Regex* matching algorithm and manually extracted age (a) from the NHLS database and (b) from the 300 random sample validation data set. The two-way mixed intraclass correlation coefficient shows agreement between manual and machine annotated age to be (a) 0.989 (CI: 0.989-0.990) and (b) 0.995 (0.994-0.996).

**Figure 5:**
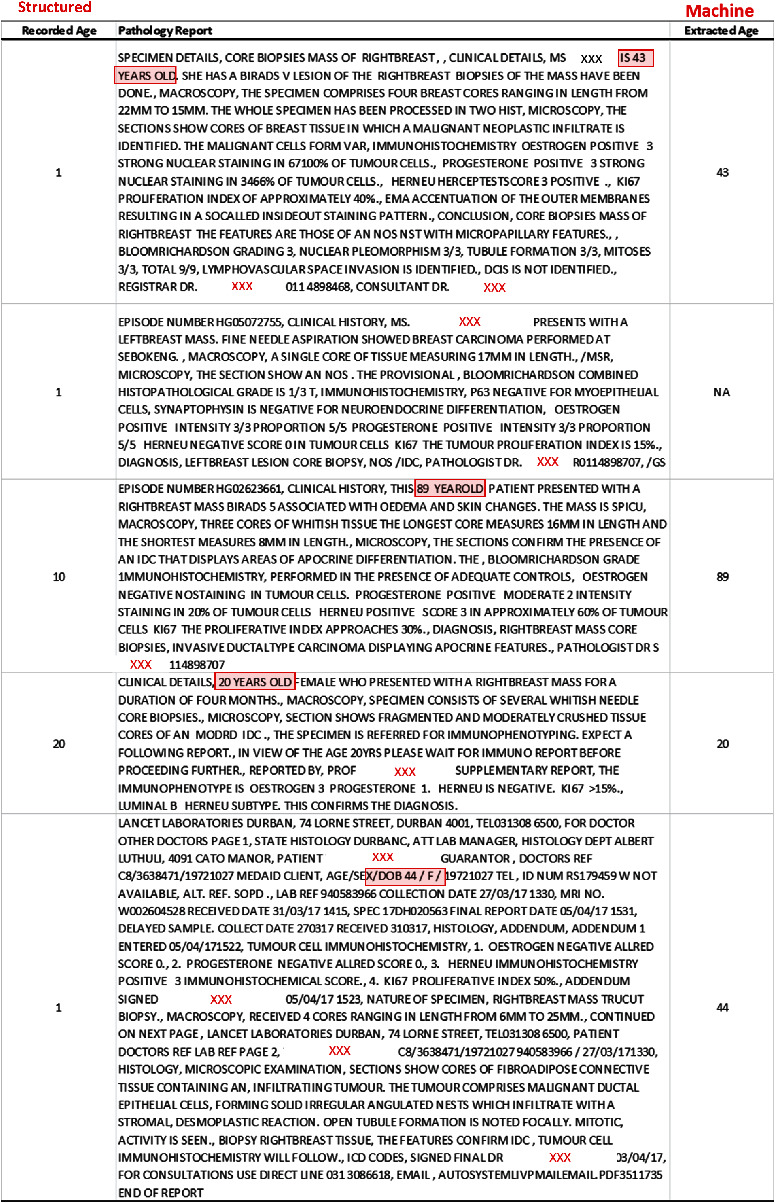
Annotation comparison between the manual and machine-assisted procedure for age using five samples. The target values are highlighted in a red box.

Figure 4 shows the relationship between the *Regex*-annotated age and the manually annotated age from the database coders and our 300 random samples. In Figure 4(a), we observed that most of the data points are clustered along the diagonal line; only a few points deviate from the diagonal. In Figure 4(b), almost all the points are on the diagonal line. These figures indicate a high agreement between our approach and the manual annotations. Further evaluation using ICC shows that performance values were 0.989 and 0.995, supporting a high performance of the rule-based extraction approach. Error analysis was conducted to assess a disagreement between the two annotators.  Figure 5 shows a sample of the error assessment between the two annotators; we observed differences between the manually-annotated age (by the NHLS-CDW data coders) and the age written in the pathology report. The rule-based approach appears to match the target parameter values more correctly than the manual annotator in these five samples. The sources of the errors are mainly from the coders, which disagrees with what is captured in the pathology report. Comparing the disagreement between the manual extraction (*N* = 300) and the machine, we observed that the machine incorrectly annotated three samples, as a result of an error in reporting of the “age” parameter (Figure S1).

Table 3 shows the summary characteristics of the study sample. The mean age was 56 ± 14.29 years. The majority (68%) of the patients diagnosed were between the age of 40 and 69 years. A large proportion of the patient had infiltrate ductal carcinoma (88%). Histological grades II and III have the highest number of observations compared to grade I. The immunohistochemistry study showed that the proportion of ER-positive was higher when compared to ER-negative. Approximately 52% (5107) of the tumours were positive for PR, while 25% were positive for HER2. The proportion of tumours with Ki67 ≥ 14 is higher than those with a low Ki67 index. Approximately 52% of the tumours are classified as Luminal A, 16% as Luminal B, 9% as HER2-OE, and 16% as TNBC. We had missing information on some parameters, including race (in a structured format). The MissForest imputation was used to impute missing data in these four parameters, and the errors were 15% and 11% for numeric and categorical parameters, respectively.

**Figure 6:**
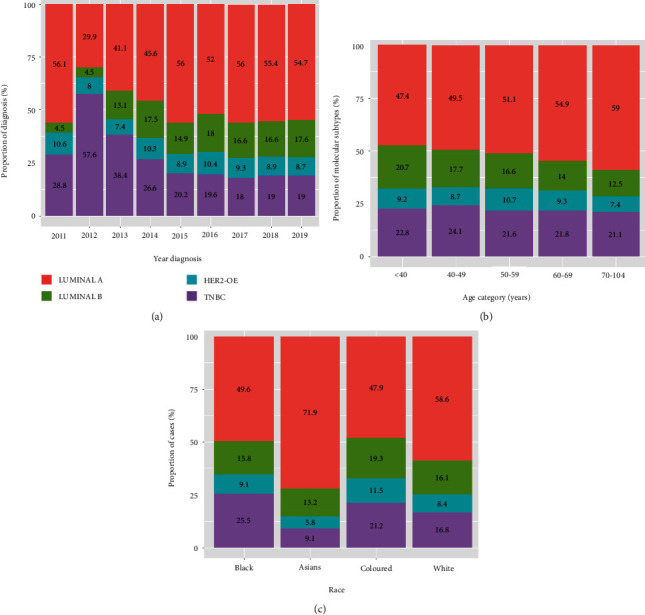
Proportion of each molecular subtype among breast cancer cases across (a) study year, (b) patient age category, and (c) racial groups.

**Figure 7:**
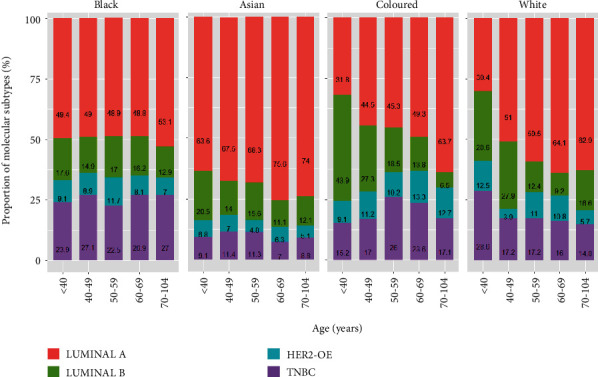
Proportion of each molecular subtype among breast cancer cases across (a) racial group and (b) patient age category.

There is no consistent pattern seen in the trend of molecular subtype over the years (Figure 6(a)). However, we observed a high proportion of Luminal A across the study years, except in 2012, where the TNBC subtype showed the highest observed incidence compared to other years. The proportion of Luminal A showed an increasing trend with an increase in patients' age, while a decreasing trend is observed in Luminal B with an increase in age (Figure 6(b)). This figure also shows that younger patients appear to have a higher proportion of TNBC and HER2-OE than older patients. In Figure 6(c), the proportion of Luminal A was high across all racial groups when compared to the other molecular subtypes, with no consistent pattern.  Figure 7 illustrates the proportion of each molecular subtype with respect to age categories by race. There is a high proportion of Luminal A for each racial group across all ages. The Asian group shows an increasing trend of Luminal A with an increase in age and a decreasing trend of Luminal B with an increase in age. The same decreasing trend of Luminal B was observed in coloured and white racial groups across the age categories. Another remarkable trend in this result shows that the Asian race across all age groups has the lowest proportion of TNBC compared to other racial groups.

The univariate relationships between the molecular subtype and other study parameters are shown in Table 4 for the imputed cases. The relationship between the molecular subtypes and other parameters in the imputed cases corresponds with the CC case analysis (Table S1 and Figure S1). Women with Luminal B were statistically less likely (0.18-0.51) to be diagnosed between the ages of 40 years and older compared to women with Luminal A subtype, while women with Basal and Her2-OE cases were only less likely to be diagnosed above 70 years and 60 years, respectively, as compared to Luminal A. Regarding Ki67, we observed that women with Luminal B, HER2-OE, and TNBC are more likely to be diagnosed with a higher Ki67 proliferation index than women with Luminal A. More specifically, these women had more than three times the odds of being diagnosed with higher Ki67 than women with Luminal A. In addition, compared to the Luminal A subtype, women with Luminal B, HER2-OE, and TNBC tended to have higher-grade tumours. We also observed that women with TNBC are less likely to be from non-black racial groups when compared to women with Luminal A. Besides, this pattern was also noted in patients with other subtypes, except for coloured women, who are more likely to be diagnosed with Luminal B and HER2-OE than Luminal A. Finally, Luminal B, HER2-OE, and TNBC were more likely to be diagnosed with non-IDC than IDC cases.

**Figure 8:**
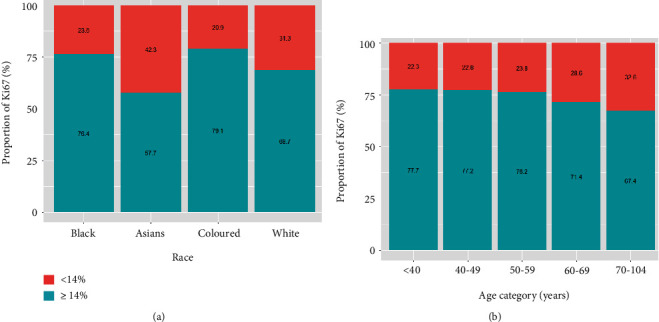
Proportion of Ki67 proliferation index among breast cancer cases across (a) patient age category and (b) racial group.

**Figure 9:**
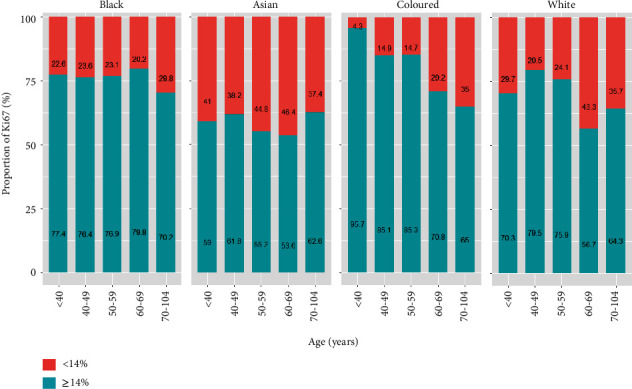
Proportion of Ki67 proliferation index among breast cancer cases by age and across racial groups.

Figure 8 presents the distribution of the Ki67 proliferation index across the racial and age groups.  Figure 8(a) shows that black and coloured women are more likely to be diagnosed with a high Ki67 proliferation index compared to Asians and white women in South Africa. There was a consistently high Ki67 (≥14%) proliferation index across all age categories, and this index negatively correlates with age (Figure 8(b)). Within each racial group, the high trend of Ki67 appears to decrease with an increase in age only in the coloured group compared to other racial groups (Figure 9). This figure also shows that Asian patients appear to have a lower proportion of Ki67 overexpression than other racial groups. The relationship between the Ki67 proliferation index and the parameters is shown in Table 5. The patterns seen in this relationship correspond to the pattern seen with the CC analysis (Table S2). Table 5 shows that patients aged 60 years and above are more likely to have a lower proliferation index compared to younger patients. Generally, the pattern shows a decrease with an increase in age and are statistically significantly from 60 years of age. There is a strong significant relationship between the Ki67 and the hormone receptors; the results show that women with a positive score for oestrogen or progesterone receptors tend to have a lower proliferation index than women with negative hormone score receptors. However, women with positive human epidermal growth factor (HER2) scores were more than twofold more likely to be diagnosed with a higher proliferation index than women with negative HER2 scores. We observed a higher odds of proliferation index for patients with tumour grades II and III than patients with grade I, with grade III showing more than 18 times the chances of higher Ki67 than grade I. Women from Asian and white racial groups were 0.32-0.58 less likely to be diagnosed with a more increased proliferation index than black women.

## 4. Discussion

Why did we focus on the molecular subtypes and Ki67 overexpression among other clinical parameters? Molecular subtypes of BC based on hormone receptors and HER2 are strong prognostic and predictive factors. Therefore, categorising BC into appropriate molecular subtypes is essential for therapeutic decision-making, vital within a population. Knowledge is scarce on the trend in Ki67 overexpression within a population. The association of Ki67 overexpression index with breast tumour outcomes has been proven both in patients experiencing chemotherapy and in patients treated with antihormonal therapy [46]. In addition to chemotherapy, some studies have shown the relationship between Ki67 and other BC prognostic parameters [4]. Therefore, it might be rational to presume that the relationship of Ki67 with BC outcome may involve a combination of prognostic and predictive effects. Hence, the trend in Ki67 overexpression in a population is highly relevant in BC epidemiology.

The spread of the extracted hormone receptors and HER2 in this study is comparable to what has been reported in the earlier study using cases from the NHLS [2]. In their study, they extracted 32%/68% ER-/ER+ (versus 34%/66% in our study), 46%/53% PR-/PR+ (versus 46%/53% in our study), and HER2-/HER2+75%/25% (versus 75%/25% in our study). In addition, we also compared the distribution of patient age, tumour grade, and race extracted in Dickens et al. [2] with our findings and found a close pattern of distribution of these parameters. A more recent study done in four South African BC units extracted some breast cancer prognostic parameters using manual extraction approach [1]. The recent study reported a mean age of 56 ± 14.4, corresponding to the mean of our system-extracted age (56 ± 14.4). Besides the corroboration in age, we also observed similarities in race and grade trends.

The distribution trend of molecular subtypes of BC was noted in Dickens et al. [1, 2] with a minor variation in pattern. The first study by Dickens et al. [2] reported that Luminal A was the most common across all races (54%-65%), followed by TNBC (17%-23%), Luminal B, and HER2-OE (8%-14%). The second study by Toma et al. [1] described the Luminal B subtype as the most common, except for a study centre, where Luminal A was the highest. The TNBC and the HER2-OE are the third and fourth in the ranking of the subtypes. Our findings of the distribution of the molecular subtypes correlate with the patterns found in the study by Dickens et al. [2]; however, these prior studies, including our study, found that HER2-OE is the least common subtype in South Africa. Our study also agrees with international studies that have explored the trend of these subtypes [5, 45]. With respect to the correlation between molecular subtype and age, our study corroborates with Dickens et al. [2], which shows that the proportion of Luminal A increased with age and showed a decreasing pattern with Luminal B, as well as HER2-OE and TNBC at an older age. Overall, our findings in the relationship between the molecular subtype and the individual clinicopathological characteristics agree with published literature showing a significant association between the molecular subtypes and other prognostic parameters.

Regardless of the inconsistency in the cut-off points and the lack of a standardised system for assessing Ki67 proliferation, identifying the predictive and prognostic values of the parameter has been regularly appealing for researchers. Hence, we postulate that the proliferation pattern of BC tumours in the South African population may inform the cancer community of its impact on treatment decision, cancer recurrence, and survival. In our study, we used 14% as the cut-off to distinguish between low expression (<14%) and high expression (≥14%) as discussed in several studies [47–49]. Previous studies have shown that the association between the Ki67 proliferation index and the other BC prognostic factors remains ambiguous and has varied across studies. Some studies have shown that Ki67 is associated with hormone receptors and HER2 [4, 49–51]. This is congruent with our study because patients with negative HR tended to have high Ki67 expression levels, while patients with a positive score for HER2 showed a high Ki67 expression index. We found that TNBC, Luminal B, and HER2-OE are more likely to have a higher Ki67 proliferation index than Luminal A; this has been shown in a study by [4]. Our study also showed that high-grade tumours were strongly associated with high expression of Ki67 [50].

Besides the methodological approach used in the extraction process, the strength of this study is using a national pathology laboratory as the only data source, which fully represents BC diagnosis across South Africa. The study data exhaustively cover the different histologic types of BC over nine years. However, there are a few limitations to this study, one of which is the lack of completeness and the intricacies in the reporting style of some of these parameters, which could have impacted the extraction process. This could have resulted in missing data for some cases. In addition, there were ambiguous cases in reporting these key parameters, especially when very long sentences were used to convey a simple message. As earlier noted, these are problems associated with free-text narrative-style reporting; hence, more study data could have been extracted if the reporting was in a synoptic style format. This has been noted in previous studies that advocated for synoptic style reporting, especially for auditing of pathology report databases [1, 7].

In conclusion, a ruled-based *Regex* NLP algorithm was proposed to extract clinically meaningful prognostic parameters from free-text BC pathology reports. Our approach achieved a high-performance measure for all the target parameters. Extracted parameters were used to explore the trend in the incidence of molecular subtypes and Ki67 and their association with other factors. This type of study helps evaluate the comprehensiveness of pathology parameter reporting and the support to encourage a synoptic or standardised report style at the national level. In addition, this type of study can be used in planning screening and diagnosis and treatment within the country. We have used BC as a case study; we encourage future studies to investigate the applicability of our proposed approach to other cancers.

## Figures and Tables

**Pseudocode 1:**
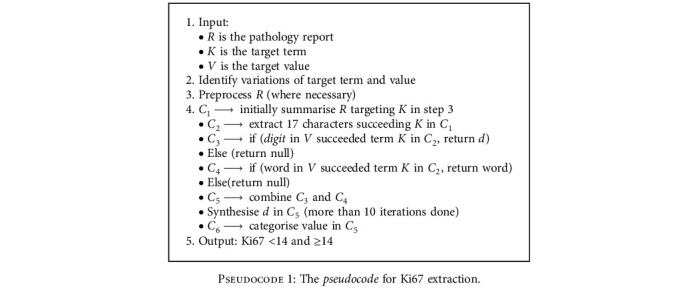
The *pseudocode* for Ki67 extraction.

**Pseudocode 2:**
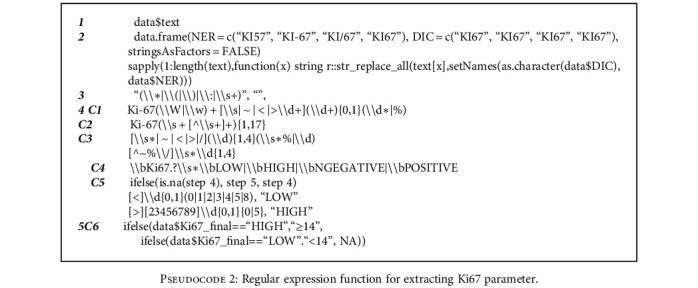
Regular expression function for extracting Ki67 parameter.

**Table 1:** Classification of the molecular subtypes.

ER	PR	HER2	Molecular subtype
+	-/+	—	Luminal A
+	-/+	+	Luminal B
—	—	+	HER2-OE
—	—	—	TNBC

**Table 2:** Evaluation of the *Regex* matching algorithm extractions on 300 random validation samples.

Parameter	Category	Precision (CI)	Recall (CI)	*F*_1_-score (CI)	Kappa (CI)
ER	Negative	0.96 (0.91-0.98)	0.93 (0.87-0.97)	0.95 (0.89-0.98)	0.92 (0.89-0.95)
	Positive	0.97 (0.94-0.98)	0.98 (0.96-0.99)	0.98 (0.95-0.99)	
PR	Negative	0.95 (0.91-0.98)	0.99 (0.96-0.99)	0.97 (0.93-0.99)	0.98 (0.96-0.99)
	Positive	0.99 (0.97-0.99)	0.96 (0.92-0.98)	0.98 (0.94-0.99)	
HER2	Negative	1.00 (0.98-1.00)	1.00 (0.99-1.00)	1.00 (0.98-1.00)	0.99 (0.97-1.00)
	Positive	1.00 (0.98-1.00)	0.99 (0.94-0.99)	0.99 (0.95-0.99)	
Ki67	<14	0.84 (0.73-0.91)	0.98 (0.90-0.97)	0.91 (0.81-0.95)	0.95 (0.91-0.97)
	≥14	0.99 (0.97-0.99)	0.94 (0.91-0.98)	0.97 (0.93-0.98)	
Grade	I	0.83 (0.60-0.93)	1.00 (0.89-1.00)	0.91 (0.91-0.98)	0.97 (0.94-0.98)
	II	0.97 (0.92-0.99)	0.97 (0.92-0.99)	0.97 (0.92-0.99)	
	III	0.99 (0.94-0.99)	0.95 (0.89-0.98)	0.97 (0.91-0.98)	
Type	IDC	1.00 (0.99-0.99)	1.00 (0.99-0.99)	1.00 (0.99-0.99)	1.00(0.99-1.00)
	Others	1.00 (0.95-1.00)	1.00 (0.95-1.00)	1.00 (0.95-1.00)	
Laterality	Left breast	0.99(0.95-0.99)	0.99 (0.96-0.99)	0.99 (0.96-0.99)	0.99 (0.95-0.99)
	Right breast	0.99 (0.96-0.99)	0.98 (0.94-0.99)	0.99 (0.95-0.99)	

**Table 3:** Description of the extracted parameters for the patients included in this study (*N* = 9669).

Variable	Category	*N*	%
Age	<40	1366	14.13
	40-49	2222	22.98
	50-59	2206	22.82
	60-69	2056	21.26
	70-104	1670	17.27
	Missing	149	1.54
Race^∗^	Asian	409	4.23
	Black	4136	42.78
	Colored	403	4.17
	White	615	6.36
	Missing	4106	42.47
Year^∗^	2011	66	0.68
	2012	311	3.22
	2013	649	6.71
	2014	870	9.00
	2015	1043	10.79
	2016	1254	12.97
	2017	1754	18.14
	2018	1829	18.92
	2019	1893	19.58
Laterality	Left breast	4481	46.34
	Right breast	4302	44.49
	Missing	886	9.16
Histologic type	IDC	8531	88.23
	Others	1138	11.77
Grade	I	610	6.31
	II	3322	34.36
	III	2806	29.02
	Missing	2931	30.31
ER	Negative	3303	34.16
	Positive	6366	65.84
PR	Negative	4562	47.18
	Positive	5107	52.82
HER2	Negative	7220	74.67
	Positive	2449	25.33
Ki67	<14	1921	19.87
	≥14	5504	56.92
	Missing	2244	23.21
Molecular subtype	Luminal A	5061	52.34
	Luminal B	1566	16.20
	HER2-OE	883	9.13
	TNBC	2159	22.33

^∗^Structured data from the NHLS database.

**Table 4:** Univariable multinomial result from the association between the clinicopathology parameters and the molecular subtype.

Parameters	Category	Luminal A	Luminal B			HER2-OE			TNBC		
		(*n* = 5061)	(*n* = 1566)	OR (95% CI)	*p* value	(*n* = 883)	OR (95% CI)	*p* value	(*n* = 2159)	OR (95% CI)	*p* value
Age	<40	647	283	1.00		125	1.00		311	1.00	
	40-49	1169	419	0.82 (0.69-0.98)	.029	206	0.91 (0.72-1.16)	0.456	569	1.01 (0.86-1.20)	0.884
	50-59	1132	367	0.74 (0.62-0.89)	.001	237	1.08 (0.85-1.37)	0.507	478	0.88 (0.74-1.04)	0.141
	60-69	1129	287	0.58 (0.48-0.70)	<0.001	192	0.88 (0.69-1.12)	0.308	448	0.83 (0.69-0.98)	0.031
	70-104	985	209	0.49 (0.40-0.60)	<0.001	123	0.65 (0.49-0.84)	0.001	353	0.75 (0.62-0.89)	0.002
Ki67	<14	2044	258	1.00		99	1.00		273	1.00	
	≥14	018	1307	3.43(2.97-3.97)	<0.001	784	5.36 (4.32-6.66)	<0.001	1886	4.68 (4.07-5.38)	<0.001
Grade	I	1062	121	1.00		30	1.00		30	1.00	
	II	2574	835	2.85 (2.32-3.45)	<0.001	305	4.18 (2.86-6.13)	<0.001	305	3.45 (2.64-4.50)	<0.001
	III	1426	609	3.75 (3.04-4.63)	<0.001	548	13.57 (9.32-19.76)	<0.001	548	17.78 (13.70-23.07)	<0.001
Laterality	Left breast	2552	765	1.00		476	1.00		1117	1.00	
	Right breast	2510	800	1.06 (0.95-1.19)	0.286	407	0.87 (0.75-1.00)	0.056	1042	0.95 (0.86-1.05)	0.306
Race	Black	3017	959	1.00		553	1.00		1549	1.00	
	Asian	608	112	0.58 (0.47-0.72)	<0.001	49	0.44 (0.32-0.60)	<0.001	77	0.25 (0.19-0.32)	<0.001
	Colored	769	310	1.27 (1.09-1.47)	0.002	185	1.31 (1.09-1.58)	0.004	341	0.86 (0.75-0.99)	0.042
	White	668	184	0.87 (0.72-1.04)	0.116	96	0.78 (0.62-0.99)	0.040	192	0.56 (0.47-0.66)	<0.001
Histologic type	IDC	4387	1403	1.00		795	1.00		1946	1.00	
	Others	75	162	0.75 (0.63-0.90)	0.002	88	0.72 (0.57-0.91)	0.006	213	0.71 (0.60-0.84)	<0.001

**Table 5:** Univariable logistic regression result from the association between the clinicopathology parameters and the Ki67 proliferation index.

Parameters	Category	<14	≥14		
		(*n* = 1918)	(*n* = 5499)	OR (95% CI)	*p* value
Age	<40	234	816		
	40-49	415	1405	0.97 (0.81-1.16)	0.750
	50-59	404	1297	0.92 (0.77-1.10)	0.377
	60-69	456	1136	0.71 (0.60-0.86)	<0.001
	70-104	409	845	0.59 (0.49-0.71)	<0.001
ER	Negative	262	1868		
	Positive	1656	3631	0.31 (0.27-0.35)	<0.001
PR	Negative	614	2592		
	Positive	1304	2907	0.53 (0.47-0.59)	<0.001
HER2	Negative	1629	3903		
	Positive	289	1596	2.3 (2.01-2.65)	<0.001
Grade	I	539	363		
	II	1154	2298	2.96 (0.54-3.44)	<0.001
	III	225	2838	18.73 (15.51-22.69)	<0.001
Laterality	Left breast	943	2777	1.00	
	Right breast	975	2722	0.95 (0.85-1.05)	0.314
Race	Black	1140	3696	1.00	
	Asian	284	387	0.42 (0.36-0.50)	<0.001
	Colored	207	785	1.17 (0.99-1.38)	0.066
	White	287	631	0.68 (0.58-0.79)	<0.001
Histologic type	IDC	1659	4947	1.00	
	Others	259	552	0.71 (0.61-0.84)	<0.001
Molecular type	Luminal A	1474	2744	1.00	
	Luminal B	212	1054	3.78 (2.97-4.87)	<0.001
	HER2-OE	77	542	2.67 (2.28-3.14)	<0.001
	TNBC	155	1159	4.02 (3.37-4.82)	<0.001

## Data Availability

Data will be made available by the authors on request.

## Supplementary Materials

Supplementary Materials Supplementary Section.pdf: description of study parameters. Error analysis of extracted age (Figure S1). Figures and tables for complete case analysis (Figure S2, Tables S1-S2). Bibliography.
