# Supplementary material for: Rule-Based Information Extraction from Free-Text Pathology Reports Reveals Trends in South African Female Breast Cancer Molecular Subtypes and Ki67 Expression
Source: Biomed Res Int. 2022 Jan 20;2022:6157861. doi: 10.1155/2022/6157861 (PMC8960023; doi:10.1155/2022/6157861)
Supplement: Supplementary Materials — Supplementary Section.pdf: description of study parameters. Error analysis of extracted age (Figure S1). Figures and tables for complete case analysis (Figure S2, Tables S1-S2). Bibliography. [file 6157861.f1.pdf]

Rule-based information extraction from free-text pathology reports reveals trends in South African female breast cancer molecular subtypes and Ki67 expression

Authors: Okechinyere J. Achilonu[1]*, Elvira Singh[1,2], Gideon Nimako[1,3], René M.J.C Eijkemans [4], Eustasius Musenge[1].

[1]Division of Epidemiology and Biostatistics, School of Public Health, Faculty of Health Sciences, University of the Witwatersrand, Parktown, Johannesburg, South Africa
[2]National Cancer Registry, National Health Laboratory Service, 1 Modderfontein Road, Sandringham, Johannesburg, South Africa
[3]Industrialization, Science, Technology and Innovation Hub, African Union Development Agency (AUDA-NEPAD), Johannesburg, South Africa
[4]Julius Center for Health Sciences and Primary Care, University Medical Center, Utrecht University, Utrecht, The Netherlands

Supplementary Section

Study parameters

Hormone receptor and human epidermal growth factor

Report on breast invasive hormone receptor and human epidermal growth factor receptor 2 (HER2) statuses are used to convey information on the prognosis of the tumour and treatment decision. Several ways of reporting were observed in the pathology reports for each of the receptors identified. These were standardised in the pre-processing phase. For the hormone receptor status graded using Allred scoring and grading system (e.g I 2/3, P 1/5), the total score was obtained by summing up the scores for the proportion of cells strained (5 scores) and the score for the intensity of staining (3 scores). The total scores range from 0 to 8; values greater than two were considered positive otherwise, negative [1]. We also classify some scores that were reported in percentages or range of percentages. Other reports were specific in mentioning if the hormone receptor was positive or negative in their phrases. We also standardised the HER2 parameter by recoding the reported values to negative, positive and equivocal [2]. The molecular subtype variables were derived following the study done by Jamshidi et al. [3]. In the presence of missing information, we extracted a proportion of the cases based on the completeness of the molecular sub-typing.

KI67

KI67 proliferative index is a valuable breast cancer marker that correlates. We also identified significant variability in the way ki67 was reported, such as "ki67 proliferation index 20%.", "ki675% staining of tumour cells", "ki-67 proliferation index nuclear staining in approximately 40%" and "ki67 60%". The variability in mentioning the term "KI67" was standardised in the pre-processing phase, while the variability in the actual values was extracted in the extraction process. The Ki67 scores range from 1 to 100, the categorisation of its values follows the study by [4–6], where two classes < 14 and ≥ 14 were formed from the values. Some of the reports containing the values of this parameter as low or poor were considered to be <14, while others with reported as high or strong, were considered to be ≥ 14.

Age

Patient age is a significant risk factor for breast cancer and has been shown to correlate with important parameters in cancer measured at diagnosis and influence survival. A possible variant in reporting patient age in the pathology reports includes "56 YEAR OLD", "AGE 31", "20YRS","AGE/SEX/ DOB 71 / F / 19470105", and "21Y" were recognised in the text. We used pattern matching in the extraction process to extract the values of the reported patient age in the database. Patient age was further categorised following studies including [7].

Race

Racial disparities have been associated with molecular sub-typing. However, the patient race was the least reported parameter observed in the database. The National Cancer Registry of South Africa uses the hot-deck imputation method to predict patient race group [7]. This has been in use for more than a decade. To predict a patient race for each pathology report, the

patient names in the pathology report are match to a reference database that contains known racial groups surnames. For some of the cases with unknown surnames in the reference database, we used the Miss-forest imputation method to predict the racial groups of these patients.

Histology grade

Grading of tumour provides essential information of the patient outcome. Nottingham system and Bloom-Richardson are used for breast cancer grading classification. For the grades describes with numbers in the pathology report, we summed the scores for the gland formation, nuclear grades and mitotic count of obtaining a total grade score ranges from 3 to 9. Grade scores of 3-5, 5-7, and 8-9 are categorised into grade I grade II and grade III, respectively [8]. For those reports with tumour grading values, such as highly differentiated, moderately differentiated and poorly differentiated, their values were recoded to Grade I, II and III [8].

Histology type

Most pathology reports included the tumour histology type; we considered extracting this parameter from the SNOMED code. We followed the procedure of xxx to map the codes to the ICD-03. This approach is likely to be more comprehensive than the direct extraction from the report. For instance, the morphology code corresponding to $M - 85003$ according to [9] is referred to as infiltrating or invasive ductal carcinoma. There are 164 categories of morphology codes; we regrouped this parameter as invasive ductal carcinoma and non-invasive ductal carcinoma. since most cases studied are in the former category

Laterality

Breast cancer laterality is a compulsory reported parameter used by pathologists to convey information on the side of the breast cancer that occurs. This study identified a few variants of names used to refer to this parameter, including “LEFT BREAST” and “LEFT, B”. These variants were standardised to LEFT BREAST and RIGHT BREAST; however, we have four cases with both left and right breast cancer, which were not considered in this study.

Year

For a report with a year of diagnosis. We identified and matched the different reporting patterns of this parameter, including ”2019/05/26” and “01-02-2014”.Although this parameter was also manually coded as a string value. We compared our extracted year with the manually coded year; we leverage their diagnosis year from the year manually coded for reports with no reported year.

Error analysis of extracted patient age

Manual		Machine
Extracted Age	Pathology report	Extracted Age
47	EPISODE NUMBER SA03422496 CLINICAL HISTORY 47YEATS FEMALE. LEFTBREAST MASS HARD SUSPECTED NONBENIGN. FOLLOW UP BREAST CLINIC MPH 28/10/19. MACROSCOPY THE SPECIMEN CONSISTS OF MULTIPLE FRAGMENTS OF TISSUE THE LARGEST MEASURES 2MM IN GREATEST DIMENSION. MICROSCOPY SECTIONS SHOW MULTIPLE FRAGMENTS OF FIBROADIPOSE TISSUE WITH MULTIPLE INVASIVE NESTS AND GLANDS OF ATYPICAL EPITHELIAL CELLS. T IMMUNOHISTOCHEMISTRY OESTROGEN POSITIVE PROGESTERONE NEGATIVE HERNEU POSITIVE KI67 40% PATHOLOGICAL DIAGNOSIS LEFTBREAST BIOPSY INVASIVE DUCT CARCINOMA REPORTED BY DR XXX	NA
22	FINAL PATIENT NAME XXX . LAB NAME CENTRAL LABORATORY LAB REF NO. 940465074 WARD GREYS/ED01789765 AGE/SEX/ DOB 21 / F / 19960518 SPEC NO. 19DH011500 NHLS HOSP RS196268 COLLECTION DATE 08/02/19 NHLS LAB MAMM RECEIVE DATE 13/02/19 0019 REPORT DATE 18/02/19 1711 REPORT FOR DOCTOR OTHER DOCTORS GUARANTOR INFORMATION STATE HISTOLOGY DURBAN C SUBMIT DR STATE HISTOLOGY DURBAN C NAME ATT LAB MANAGER CONTACT NO. H HISTOLOGY DEPT ALBERT LUTHULI EMAIL CATO MANOR 4091 MEDAID CLIENT DELAYED SAMPLE. COLLECT DATE 080219 RECEIVED 130219 ADDENDUM ADDENDUM 1 ENTERED 18/02/191423 HORMONE RECEPTOR STATUS OESTROGEN STAINING POSITIVE INTENSITY 2 DISTRIBUTION 3 66% 100% OF CELLS SCORE 5 4 6 HIGH SCORE PROGESTERONE STAIN RESULT POSITIVE HERNEU ONCOGENE EXPRESSION SCORE 0 NEGATIVE KI67 PROLIFERATION INDEX INTERMEDIATE 20% OF NUCLEI STAIN POSITIVELY ADDENDUM SIGNED XXX 18/02/19 1529 CONTINUED ON NEXT PAGE FINAL PAGE 1 PATIENT NAME XXX LAB NAME CENTRAL LABORATORY LAB REF NO. 940465074 DR. REF NO. GREYS/ED01789765 AGE/SEX/ DOB 22 / F / 19960518 SPEC NO. 19DH011500 ID NUM. RS196268 COLLECTION DATE 08/02/19 CONTACT NUM/S H C RECEIVE DATE 13/02/19 0019 EMAIL . REPORT DATE 18/02/19 1711 DELAYED SAMPLE. COLLECT DATE 080219 RECEIVED 130219 NATURE OF SPECIMEN BIOPSY OF LEFTBREAST . CLINICAL HISTORY 22 YEAROLD PATIENT WITH LOBULATED MASS OF LEFTBREAST . CORE BIOPSY SUBMITTED FOR HISTOLOGICAL EXAMINATION. MACROSCOPY SPECIMEN SUBMITTED WAS COMPRISED OF 2 CORES OF TISSUE LONGER OF WHICH MEASURED 1.2 CM IN LENGTH. TOTAL SAMPLE PROCESSED AND SERIAL SECTIONS CUT. MICROSCOPIC EXAMINATION PATHOLOGY OBSERVED IS DETAILED IN CONCLUSION BELOW. BIOPSY OF LEFTBREAST MASS 2 CORES 1. INFILTRATING DUC CARCINOMA. 2. MICROSCOPIC GRADE 2 NOTTINGHAM SYSTEM. 3. NO INSITU COMPONENT OR SECONDARY GROWTH PATTERN PRESENT. 4. VASCULAR INVASION NOT OBSERVED. 5. HORMONE RECEPTOR RESULTS TO FOLLOW. ICD CODES SIGNED FINAL DR XXX 14/02/19 FOR CONSULTATIONS USE DIRECT LINE 031 3086510 EMAIL AUTOSYSTEMLIVPMAILKZNEMAIL.PDF1843413 END OF REPORT	21
47	EPISODE NUMBER SA03422 49 Y 47 YEAOLD FEMALE. LEFTBREAST MASS HARD SUSPECTED NONBENIGN. FOLLOW UP BREAST CLINIC MPH 28/10/19. MACROSCOPY THE SPECIMEN CONSISTS OF MULTIPLE FRAGMENTS OF TISSUE THE LARGEST MEASURES 2MM IN GREATEST DIMENSION. MICROSCOPY SECTIONS SHOW MULTIPLE FRAGMENTS OF FIBROADIPOSE TISSUE WITH MULTIPLE INVASIVE NESTS AND GLANDS OF ATYPICAL EPITHELIAL CELLS. T IMMUNOHISTOCHEMISTRY OESTROGEN POSITIVE PROGESTERONE NEGATIVE HERNEU POSITIVE KI67 40% PATHOLOGICAL DIAGNOSIS LEFTBREAST BIOPSY INVASIVE DUCT CARCINOMA REPORTED BY DR XXX	49

Figure S1: Comparison of annotation disagreement between manual (N=300) and machine assisted extraction for age. All the samples for age were correctly extracted by the machine except for these three samples. The target values are highlighted, although the last sample includes the machine extracted age.

Figure and Tables for the complete case analysis

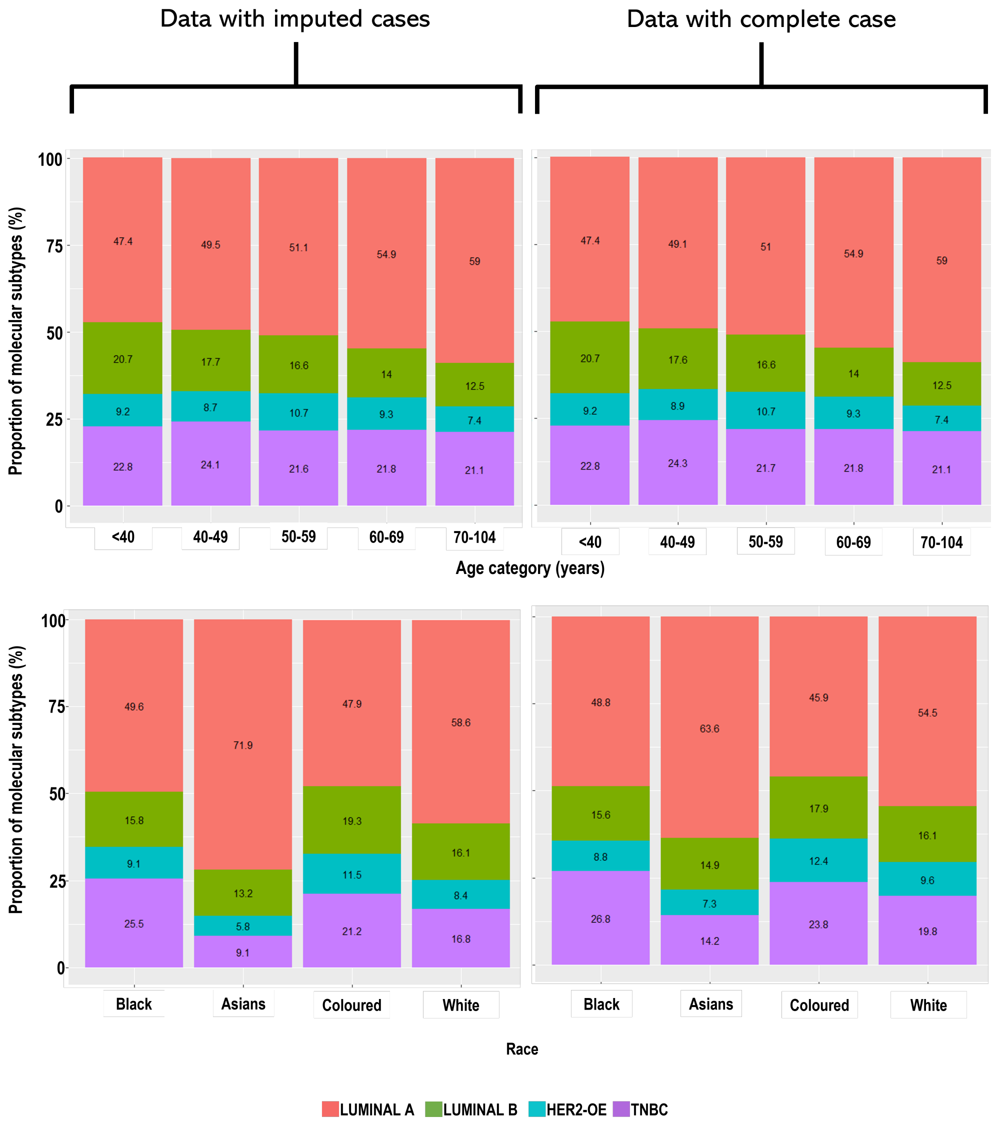

Figure S2: Proportion of of each molecular subtype among breast cancer cases across patient age category and racial group

Table S1: Univariable multinomial result from the association between the clinicopathology parameters and the molecular subtype with the complete case data

Parameters	Category	Luminal A	Luminal B			HER2-OE			TNBC		
		(n=5061)	(n=1566)	OR (95%CI)	p-value	(n=883)	OR (95%CI)	p-value	(n=2159)	OR (95%CI)	p-value
Age	<40	647	283			125			311		
	40-49	1092	391	0.82 (0.68-0.98)	0.030	198	0.94 (0.74-1.20)	0.610	541	1.03 (0.87-1.22)	0.727
	50-59	1124	367	0.75 (0.62-0.90)	0.002	237	1.09 (0.86-1.38)	0.470	478	0.88 (0.74-1.05)	0.164
	60-69	1129	287	0.58 (0.48-0.70)	<0.001	192	0.88 (0.69-1.12)	0.307	448	0.83 (0.69-0.98)	0.031
	70-104	985	209	0.49 (0.40-0.60)	<0.001	123	0.65 (0.49-0.84)	0.001	353	0.75 (0.62-0.89)	0.002
Ki67	<14	1474	214			78			155		
	≥14	2746	1056	2.65 (2.26-3.11)	<0.001	541	3.72 (2.91-4.76)	<0.001	1161	4.02 (3.36-4.81)	<0.001
Histologic grade	I	457	76			22			55		
	II	1971	581	1.77 (1.37-2.30)	<0.001	269	2.84(1.81-4.43)	<0.001	501	2.11 (1.57-2.84)	<0.001
	III	1002	405	2.43 (1.86-3.18)	<0.001	343	7.11 (4.56-11.10)	<0.001	1056	8.76 (6.53-11.74)	<0.001
Laterality	Left breast	2342	715			418			1006		
	Right breast	2266	719	1.04 (0.92-1.17)	0.525	383	0.95 (0.82-1.10)	0.481	934	0.96 (0.86-1.07)	0.446
Race	Black	2017	645	1.00		366	1.00		1108	1.00	
	Asian	260	61	0.73 (0.55-0.98)	0.038	30	0.64 (0.43-0.94)	0.024	58	0.41 (0.30-0.54)	<0.001
	Colored	185	72	1.22 (0.91-1.62)	0.179	50	1.49 (1.07-2.08)	0.019	96	0.94 (0.73-1.22)	0.664
	White	335	99	0.92(0.73-1.18)	0.521	59	0.97 (0.72-1.31)	0.844	122	0.66 (0.53-0.83)	<0.001
Histologic type	IDC	4386	1404	1.00		795	1.00		1946	1.00	
	Others	675	162	0.75 (0.63-0.90)	0.002	88	0.72 (0.57-0.91)	0.006	213	0.71 (0.60-0.84)	<0.001

Table S2: Univariable logistic regression result from the association between the clinicopathology parameters and the Ki67 proliferation index with the complete case data

Parameter	Category	<14 (n=1918)	**≥14** (n=5499)	OR (95%CI)	p-value
**Age**	<40	234	816	1	
	40-49	397	1330	0.96 (0.80-1.15)	0.669
	50-59	404	1296	0.92 (0.77-1.11)	0.372
	60-69	456	1136	0.71 (0.60-0.86)	<0.001
	70-104	409	845	0.59 (0.49-0.71)	<0.001
ER	Negative	262	1868	1.00	
	Positive	1656	3631	0.31 (0.27-0.35)	<0.001
PR	Negative	614	2592	1.00	
	Positive	1304	2907	0.53 (0.47-0.59)	<0.001
Herneu	Negative	1629	3903	1	
	Positive	289	1596	2.30 (2.01- 2.65)	<0.001
Histologic grade	I	256	231	1.00	
	II	883	1852	2.32 (1.91-2.82)	<0.001
	III	200	1847	10.23 (8.13-12.88)	<0.001
Laterality	Left breast	888	2531	1.00	
	Right breast	886	2416	0.96 (0.86-1.07)	0.425
Race	Black	707	2298	1.00	
	Asian	122	186	0.47 (0.37-0.60)	<0.001
	Colored	58	205	1.09 (0.80-1.47)	0.588
	White	132	332	0.77 (0.62-0.96)	0.021
Histologic type	IDC	1659	4947	1.00	
	Others	259	552	0.53(0.47-0.59)	<0.001

Bibliography

[1] Asim Qureshi and Shahid Pervez. Allred scoring for er reporting and it's impact in clearly distinguishing er negative from er positive breast cancers. *Journal Pakistan Medical Association*, 60(5):350, 2010.

[2] Allen M Gown. Current issues in er and her2 testing by ihc in breast cancer. *Modern pathology*, 21(2):S8–S15, 2008.

[3] Neema Jamshidi, Shota Yamamoto, Jeffrey Gornbein, and Michael D Kuo. Receptor-based surrogate subtypes and discrepancies with breast cancer intrinsic subtypes: implications for image biomarker development. *Radiology*, 289(1):210–217, 2018.

[4] Nahed A Soliman and Shaimaa M Yussif. Ki-67 as a prognostic marker according to breast cancer molecular subtype. *Cancer biology & medicine*, 13(4):496, 2016.

[5] Fabinshy Thangarajah, Insa Enninga, Wolfram Malter, Stefanie Hamacher, Birgid Markiefka, Lisa Richters, Stefan Kraemer, Peter Mallmann, and Verena Kirn. A retrospective analysis of ki-67 index and its prognostic significance in over 800 primary breast cancer cases. *Anticancer research*, 37(4):1957–1964, 2017.

[6] Soomin Ahn, Junghye Lee, Min-Sun Cho, Sanghui Park, and Sun Hee Sung. Evaluation of ki-67 index in core needle biopsies and matched breast cancer surgical specimens. *Archives of pathology & laboratory medicine*, 142(3):364–368, 2018.

[7] Caroline Dickens, Raquel Duarte, Annelle Zietsman, Herbert Cubasch, Patricia Kellett, Joachim Schüz, Danuta Kielkowski, and Valerie McCormack. Racial comparison of receptor-defined breast cancer in southern african women: Subtype prevalence and age–incidence analysis of nationwide cancer registry data. *Cancer Epidemiology and Prevention Biomarkers*, 23(11):2311–2321, 2014.

[8] Leslie W Dalton, Sarah E Pinder, Christopher E Elston, Ian O Ellis, David L Page, William D Dupont, and Roger W Blamey. Histologic grading of breast cancer: linkage of patient outcome with level of pathologist agreement. *Modern pathology*, 13(7):730–735, 2000.

[9] Toral Gathani, Diana Bull, Jane Green, Gillian Reeves, and Valerie Beral. Breast cancer histological classification: agreement between the office for national statistics and the national health service breast screening programme. *Breast Cancer Research*, 7(6):1–7, 2005.
